# Accounting for predator species identity reveals variable relationships between nest predation rate and habitat in a temperate forest songbird

**DOI:** 10.1002/ece3.9411

**Published:** 2022-10-12

**Authors:** Nino Maag, John W. Mallord, Malcolm D. Burgess, Shannon Lüpold, Andrew Cristinacce, Raphaël Arlettaz, Sandro Carlotti, Tony M. Davis, Alex Grendelmeier, Christopher J. Orsman, Michael Riess, Pablo Stelbrink, Gilberto Pasinelli

**Affiliations:** ^1^ Swiss Ornithological Institute Sempach Switzerland; ^2^ RSPB Centre for Conservation Science Sandy Bedfordshire UK; ^3^ Division of Conservation Biology, Institute of Ecology and Evolution University of Bern Bern Switzerland; ^4^ Butterfly Conservation Southampton UK; ^5^ Department of Biology University of Marburg Marburg Germany; ^6^ Department of Evolutionary Biology and Environmental Studies University of Zürich Switzerland

**Keywords:** habitat fragmentation, nest survival, spatial scale, species specific, woodland

## Abstract

Nest predation is the primary cause of nest failure in most ground‐nesting bird species. Investigations of relationships between nest predation rate and habitat usually pool different predator species. However, such relationships likely depend on the specific predator involved, partly because habitat requirements vary among predator species. Pooling may therefore impair our ability to identify conservation‐relevant relationships between nest predation rate and habitat. We investigated predator‐specific nest predation rates in the forest‐dependent, ground‐nesting wood warbler *Phylloscopus sibilatrix* in relation to forest area and forest edge complexity at two spatial scales and to the composition of the adjacent habitat matrix. We used camera traps at 559 nests to identify nest predators in five study regions across Europe. When analyzing predation data pooled across predator species, nest predation rate was positively related to forest area at the local scale (1000 m around nest), and higher where proportion of grassland in the adjacent habitat matrix was high but arable land low. Analyses by each predator species revealed variable relationships between nest predation rates and habitat. At the local scale, nest predation by most predators was higher where forest area was large. At the landscape scale (10,000 m around nest), nest predation by buzzards *Buteo buteo* was high where forest area was small. Predation by pine martens *Martes martes* was high where edge complexity at the landscape scale was high. Predation by badgers *Meles meles* was high where the matrix had much grassland but little arable land. Our results suggest that relationships between nest predation rates and habitat can depend on the predator species involved and may differ from analyses disregarding predator identity. Predator‐specific nest predation rates, and their relationships to habitat at different spatial scales, should be considered when assessing the impact of habitat change on avian nesting success.

## Graphical abstract

Studies about relationships between nest predation rate and habitat usually pool different predator species. However, such relationships likely depend on the specific predator involved, partly because habitat requirements vary among predator species. We show that accounting for predator species identity reveals variable relationships between nest predation rate and habitat in wood warblers.

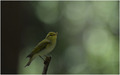

## 1 INTRODUCTION

Nest predation is the most important cause of breeding failure in ground‐nesting birds and can have important implications for their population dynamics (Newton, 1998; Ricklefs, 1969; Roodbergen et al., 2012). Nest predation rates can be affected by the extent and spatial arrangement of different habitat types (Chalfoun et al., 2002), which should be considered in the assessments of avian reproduction (Chalfoun & Martin, 2009; Thompson, 2007). For a given area of habitat, a more fragmented landscape has more smaller habitat patches with a greater total edge length and shape complexity (Ewers & Didham, 2006; Fahrig, 2017). For habitat specialists, a reduction in the area of their utilized habitat may increase nest predation through increases in predator density and the length of edge habitats in small patches (Bayne & Hobson, 1997; Stephens et al., 2004). A greater length in habitat edge may increase nest predation by predators that typically forage along edges and in multiple habitats (Andren, 1992; Lahti, 2001).

The composition of the habitat matrix (e.g., pastures, arable land; hereafter matrix) between patches of utilized habitat (e.g., forest) can also affect nest predation rates, if similarities in patch and matrix habitats lead to spillover of predator species (Cook et al., 2002; Ruffell et al., 2017) or if predator‐rich matrix habitat (e.g., agriculture) causes stronger edge effects (Andren, 1992; Dijak & Thompson, 2000). Matrix effects appear most prevalent in small patches and in edge habitats and can therefore be confounded by habitat area or edge length/complexity (Ewers & Didham, 2006). Hence, to disentangle matrix effects from area and edge effects, the proportion of different habitat types within the matrix should be quantified (Ewers & Didham, 2006; Rodewald, 2003). The effects of proportional matrix components have been investigated for bird abundance and diversity (Renjifo, 2001; Ruffell et al., 2017), but not for nest predation rates.

Even if habitat features such as habitat area, edge complexity, and matrix are quantified appropriately, identifying links between nest predation rates and these features may be impaired if predator species causing nest failures are pooled in analyses, because habitat requirements vary among predator species. Predator‐specific studies have been more successful in identifying relationships between nesting success and habitat change (Benson et al., 2010; Cox et al., 2012a; DeGregorio et al., 2014; Rodewald & Kearns, 2011) than nonpredator‐specific studies, which often find no relationships or contrasting results (Chiavacci et al., 2018). The few predator‐specific studies show that the influence of certain predators on nesting success varies considerably across landscape contexts. For example, while some raptors (e.g., red‐shouldered hawk *Buteo lineatus*, broad‐winged hawk *B. platypterus*) depredate nests along habitat edges between forest and agriculture/other habitat (Benson et al., 2010; Cox et al., 2012a, 2012b), other raptors (e.g., red‐tailed hawk *B. jamaicensis*) do not depredate nests along edge structures such as roads and power lines (DeGregorio et al., 2014). Both natural and human edge structures can be used as perches by raptors to hunt from (Meunier et al., 2000), but it is difficult to make inferences about the effects of different structures on nest predation based on few studies.

A ground‐nesting forest songbird that experiences high nest predation by multiple predator species is the wood warbler *Phylloscopus sibilatrix*, a Palearctic migrant with a European breeding range (Keller et al., 2020). Among the most important wood warbler nest predators are Eurasian jay *Garrulus glandarius*, common buzzard *Buteo buteo*, Eurasian sparrowhawk *Accipiter nisus*, pine marten *Martes martes*, red fox *Vulpes vulpes*, and European badger *Meles meles* (Bellamy et al., 2018; Grendelmeier et al., 2018; Mallord et al., 2012; Maziarz et al., 2019). Wood warbler nest predation rates are similar across its range, but avian predation is more frequent in the United Kingdom, while mammals are more important nest predators in mainland Europe (Maag et al., 2022). Previous studies have investigated relationships between wood warbler nest survival and habitat structures including nest concealment or shrub cover at the scale of the nest site or territory (Bellamy et al., 2018; Grendelmeier et al., 2015; Maziarz et al., 2019). It is unknown, however, whether predator‐specific predation rates of wood warbler nests vary across regions and whether specific rates relate to habitat type. As nest success is regulated by nest predators that differ in foraging range, the key to detect relationships between predation rate and habitat is to consider different spatial scales (Chalfoun et al., 2002; Chiavacci et al., 2018; Stephens et al., 2004).

Here, we assessed wood warbler nest predation rates by jay, buzzard, sparrowhawk, marten, fox, and badger to determine whether predator‐specific predation rates were related to forest area and forest edge complexity, and the proportion of grassland, arable land, and urban habitat in the adjacent matrix. We compared the predator‐specific rates to the pooled predation rate, which we related to the same habitat features. We tested the relationships between nest predation rates and habitat features at the local (1000 m radius around each nest) and landscape scale (10,000 m radius) in five study regions distributed across the western half of the species' breeding range (UK, Germany, Switzerland). The overall expectation was that modeling nest predation separately for each predator species would reveal more/different relationships between predation rate and habitat compared to using a pooled predation rate. Specifically, we expected predation rates by habitat generalists like buzzard (Walls & Kenward, 2020) and fox (Kurki et al., 1998) to increase with increasing edge complexity and proximity to grassland and predation rates by forest specialists like jay (Andren, 1992), sparrowhawk (Götmark & Post, 1996), marten (Kurki et al., 1998), and badger (Balestrieri et al., 2009) to increase with increasing forest area.

## 2 METHODS

### 2.1 Study areas

The fates of 559 wood warbler nests were monitored with nest cameras in five study regions distributed across the western half of the species breeding range (Figure S1): mid‐Wales, UK (52° 8’ N, −3° 45’ W, 2009–2011, *n* = 73); Dartmoor, UK (50° 34’ N, −3° 47’ W, 2012 and 2013, *n* = 65); New Forest, UK (50° 52’ N, −1° 38’ W, 2012 and 2013, *n* = 45); Hessen, Germany (50° 57’ N, 8° 55′ E, 2015 and 2020, *n* = 89); and Solothurn‐Baselland, Switzerland (47° 23’ N, 7° 35′ E, 2010–2020, *n* = 287). Wood warbler habitat varied among the study regions with respect to forest area, forest edge complexity, and adjacent matrix type (Figures S2–S4). In the United Kingdom, study regions consisted of small and scattered forest patches within a matrix of mostly grassland (pastures and natural grassland); in Germany and Switzerland, study regions consisted of large but discontinuous forest areas interspersed by a matrix of arable areas and grassland, and a small proportion of urban area. Details of forest structure in the different study regions are described in Bellamy et al. (2018), Pasinelli et al. (2016), and Stelbrink et al. (2019).

### 2.2 Nest monitoring

Surveys to locate wood warbler territories (i.e., singing males) lasted from male arrival in mid‐April to the end of the breeding season in mid‐July. Once males were paired, females were closely observed to locate nests. Nest cameras were deployed during the nest‐building or incubation stage, and then redeployed at other nests at any stage to maximize the number of nests monitored by cameras. Cameras used in the United Kingdom were custom‐built (Bolton et al., 2007) and deployed at 0.5–1.5 m from the nests (Bellamy et al., 2018; Mallord et al., 2012). In Germany and Switzerland, Reconyx trail cameras (Reconyx, Inc.) were used and deployed at 1–2 m from nests (Grendelmeier et al., 2015). Both camera types were motion triggered and produced strings of still pictures in rapid sequence.

Using camera footage and regular nest visits (usually every 1–6 days, Grendelmeier et al., 2015; Mallord et al., 2012), we estimated first egg laying date, egg hatching date, and date of failure or fledging, respectively. First egg laying dates were determined either directly for nests found before or during egg laying or for nests found later by back‐calculating based on the hatching date or developmental stage of the chicks (Grendelmeier et al., 2015; Mallord et al., 2012). On average, hatching occurred 19 days (= 6 days of egg laying +13 days of incubation) and fledging 33 days after the first egg laying date (= 1 day of hatching +13 days nestling period, Glutz von Blotzheim & Bauer, 1991). All nests from which at least one young fledged were categorized as successful nests, including partially depredated nests. All nest predators recorded by cameras are listed in the Table S1.

### 2.3 Forest area and edge

We assessed forest area and edge at two spatial scales: inside circles with radii of 1000 m for the local scale and 10,000 m for the landscape scale, respectively, around each nest. These scales have previously been shown as relevant for examining relationships between nesting success and habitat variables (Chalfoun et al., 2002; Stephens et al., 2004). For forest area, we used the total forest area (m^2^) within each circle divided by the total area of the circle, giving the proportion of forest area within each circle. For calculating forest edge length/complexity within circles, we used the fractal dimension index (FDI), which was quantified as two times the natural logarithm of the total forest edge length (m) divided by the natural logarithm of total forest area (m^2^). The FDI typically ranges between 1 and 2, with lower values indicating simple/more straight habitat edges and higher values indicating more complex edges (McGarigal, 1995). For our study areas, FDI should provide a more meaningful estimate of fragmentation than, for example, number of habitat patches in a landscape (Fahrig, 2017) due to the connectedness of forest areas in Germany and Switzerland (Figure S2). Note that FDI generally increased with decreasing forest area (see study regions in the United Kingdom and Germany, Figure S3), but in Switzerland, FDI increased with increasing forest area due to the specific configuration of forest areas (i.e., large forest areas were interspersed by patches of open areas, leading to complex edges, Figure S3).

### 2.4 Habitat matrix variables

We assessed the nonforest habitat matrix adjacent to forest areas only at the landscape scale (10,000 m circle) as 1000 m circles often only included a small amount of nonforest habitat. We categorized the matrix content into three types: grassland, arable land, and urban. Grassland mostly consisted of pasture and natural grassland, and included small fractions of heathland, sparse vegetation, and peat bogs. Arable land consisted of arable fields and mosaics of small cultivated land parcels with different cultivation types (e.g., grains, corn, rapeseed, vegetables, or fruits). Overall, grassland and arable land made up approximately 80% of the matrix, the remaining 20% consisted of discontinuous urban habitat (Figure S4). For each of the three matrix habitat types, we divided the total area (m^2^) by the total area of nonforest matrix habitat in each circle, giving the proportion of each habitat type within the matrix. We used the CORINE land cover data of Europe (CORINE Land Cover, 2013) in the software QGIS (QGIS.org, 2021) to calculate forest and matrix habitat variables.

### 2.5 Statistical analysis

We first estimated the pooled daily nest predation rate of all predator species and related it to forest habitat features and adjacent nonwoodland matrix habitat variables. Nests that were lost due to other failures (e.g., desertion, trampling, *n* = 43 nests, Table S1) were excluded from the analysis. We then assessed the relationship between the six single‐species daily nest predation rates (jay *n* = 68 nests, buzzard *n* = 16, sparrowhawk *n* = 13, marten *n* = 41, fox *n* = 18, badger *n* = 18) and forest and matrix variables. For the remaining 16 predator species identified (total of *n* = 38 nests, Table S1), small sample sizes resulted in unreliable single‐species models and we did not pursue them. In the single‐species models, these 38 nests were right censored on the day of predation by treating them as nests that “left the trial” (Fox & Weisberg, 2011). We left‐censored nests found after first egg laying by including them only from the day they were found (*n* = 327) and right‐censored nests that fledged successfully by treating them as “still alive” on the day of fledging (*n*=263; Fox & Weisberg, 2011).

We performed seven Cox hazard mixed effects models using the library *coxme* Therneau, (2020) in R, version 4.1.2 (R Core Team, 2021), one for the pooled rate and six for the single‐species rates. Each model included seven fixed effects: proportion of forest at the 1000 m and 10,000 m scale, FDI at the 1000 m and 10,000 m scale, and proportion of grassland, arable land, and urban area in the matrix. We standardized variables by subtracting their mean and dividing by their standard deviation. Due to the strong negative correlation between grassland and arable land (Pearson's correlation coefficient = −0.90, Figure S5), we made two sets of models, one set including grassland and the other set including arable land, resulting in a total of 14 models. We calculated variance inflation factors for the remaining variables to examine collinearity among them (Belsley et al., 2005). VIFs were smaller than 5, indicating that collinearity was not an issue. Each model included a random effect for study year.

Initially, we included study region as a fixed effect and tested the interactions between each habitat variable and study region to assess if the predation rates of different nest predators varied in their response to forest area and edge complexity depending on the study region. For sparrowhawk, fox, and badger, the models did not converge due to small sample sizes. For jay, buzzard, and marten, the interactions were dropped during model selection. We therefore dropped the interaction terms from all models and used study region as another random effect. Standard deviation and variance of random effects are reported in Table 1.

**TABLE 1:** Relationships between hazard rates (i.e., daily nest predation rates) and habitat variables for pooled and single‐species analyses

Model	Fixed	Coef	*β*	SE		Random	SD	Var
Pooled								
Area 1000 + Grassland	Area 1000	0.20	1.23	0.08		Year	0.27	0.07
	Grassland	0.18	1.20	0.08		Region	0.02	0.00
Area 1000 + Arable land	Area 1000	0.21	1.24	0.08		Year	0.25	0.06
	Arable land	−0.20	0.82	0.08		Region	0.01	0.00
Jay								
Null						Year	0.46	0.21
						Region	0.25	0.06
Buzzard								
Area 1000 + Area 10,000	Area 1000	0.57	1.76	0.29		Year	1.10	1.21
	Area 10,000	−1.21	0.30	0.31		Region	0.02	0.00
Sparrowhawk								
Area 1000	Area 1000	−0.56	0.57	0.32		Year	1.15	1.33
						Region	0.02	0.00
Null						Year	0.46	0.21
						Region	0.25	0.06
Marten								
Area 1000 + FDI 10,000	Area 1000	0.73	2.07	0.26		Year	0.93	0.87
	FDI 10,000	1.82	6.15	0.32		Region	0.02	0.00
Fox								
Null						Year	0.40	0.16
						Region	0.32	0.10
Badger								
Area 1000 + Grassland	Area 1000	0.50	1.66	0.24		Region	0.02	0.00
	Grassland	0.47	1.61	0.25		Year	0.24	0.06
Area 1000 + Arable land	Area 1000	0.55	1.74	0.25		Region	0.02	0.00
	Arable land	−0.48	0.62	0.25		Year	0.24	0.06

*Note*: We present the most parsimonious models identified by model selection (Tables S2, S3). Fixed effects are forest area (area) and edge complexity (FDI) at the 1000 m (local) and 10,000 m (landscape) scale, respectively, and proportion of grassland and arable land in the matrix. The coefficient (Coef), exponentiated coefficient (*β*), and standard error of the coefficient (SE) are reported for fixed effects. The standard deviation (SD) and variance (Var) are reported for random effects.

We performed model selection based on Akaike's information criterion for small sample sizes (AICc) using the library *MuMin* (Bartoń, 2018) to identify the variables best explaining daily predation rates. Where no single model was clearly identified as the most parsimonious (i.e., ΔAICc <2), we chose the model with the fewer number of parameters, as it is reasonable to conclude that a covariate is not informative if it does not improve model fit by >2 AIC units (Arnold, 2010). We performed model selection separately for models with the variables grassland (Table S2) and arable land (Table S3), and often the same variables were identified to best explain daily predation rates in the grassland and arable land models. If different variables were identified to be most important in the grassland and arable land models, we present both outputs (Table 1).

Pine martens were not among the predators of wood warbler nests in the United Kingdom (Table S1). Despite reintroductions, the pine marten is still very rare in England and Wales following near extinction due to persecution and habitat loss in the 19th century (Langley & Yalden, 1977; Stringer et al., 2018). Hence, we excluded the UK study regions (mid‐Wales, Dartmoor, New Forest) from the pine marten predation rate analysis to avoid a bias toward nonpredation by martens in UK habitats.

In the Cox hazard analysis, the hazard rate h[t] represents the rate of hazard for a given time step and was calculated at daily intervals, which in our case is the daily nest predation rate. The hazard rate is interpreted as the instantaneous rate of occurrence of nest predation (coded as 1 on the last day) in nests remaining at risk (coded as 0). The exponentiated coefficients (*β*) reported in the Cox model (Table 1) can be interpreted as the multiplicative effect of each explanatory variable on the hazard rate, that is, the relative influence of a variable on the daily nest predation rate Therneau, (2020). If the model coefficient (Coef, Table 1) is negative, then 0 < *β* < 1; if the model coefficient is positive, then *β* > 1.

**FIGURE 1:**
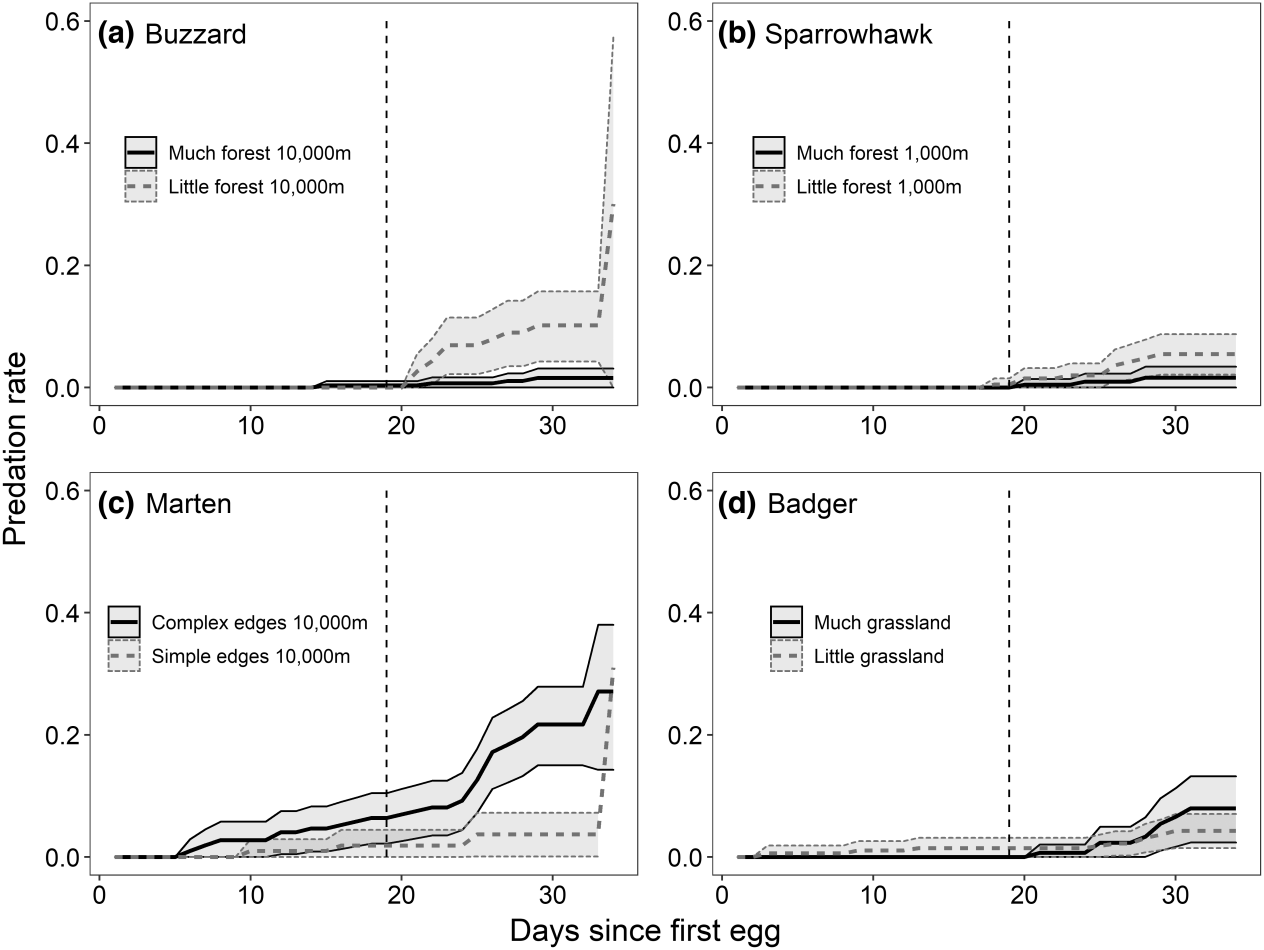
Predator‐specific daily predation rates of wood warbler nests in relation to habitat variables: little (≤ mean) and much (> mean) forest area, simple (≤ mean) and complex (> mean) forest edges, little (≤ mean) and much (> mean) grassland in the adjacent matrix. Shown are daily nest predation rates and 95% confidence intervals starting at the first egg laying date. Vertical lines indicate the average hatching date.

Because visualization of predictions is not possible in the library *coxme*, we used the library *survival* (Therneau, 2021) to produce daily nest predation rate curves (Figure 1) and predict overall nest predation by predators (Table 2). The overall predation estimates correspond to the model predictions on day 33 after egg laying (Figure S6), the average length of the nesting period in wood warblers (see above). However, the library survival does not permit the inclusion of random effects and, hence, results in Figure 1 and Table 2 represent averages across all years and regions. In addition, because the *x*‐axis represents time [t] in Cox hazard graphs (Figure 1), continuous variables must be categorized for the purpose of visualization. Therefore, we categorized forest area into little (≤ mean) and much (> mean) forest, forest edge complexity (FDI) into simple (≤ mean) and complex (> mean) edges, and grassland in the adjacent matrix into little (≤ mean) and much (> mean) grassland.

**TABLE 2:** Mean nest predation rates by predator species. Reported are number of nests (*N*), model predictions (predation rate), and 95% confidence intervals (2.5 and 97.5 CI).

Nest predator	*N*	Predation rate	2.5 CI	97.5 CI
Pooled	255	0.547	0.492	0.597
Jay	68	0.194	0.147	0.239
Buzzard	16	0.040	0.020	0.060
Sparrowhawk	13	0.035	0.016	0.054
Marten	41	0.148	0.104	0.190
Fox	18	0.056	0.030	0.082
Badger	18	0.057	0.029	0.083

*Note*: Rates give the predicted probability of predation over an entire nesting period averaging 33 days across all study regions.

## 3 RESULTS

### 3.1 Pooled nest predation

The pooled daily nest predation rate (i.e., hazard rate) was higher in locations with large local forest area (1000 m circle) and higher where the proportion of grassland in the adjacent matrix was high but arable land low (Table 1). In general, grassland and arable land complemented each other in the habitat matrix, that is, proportion of grassland increased if arable land decreased and vice versa (Figure S4), which is reflected by opposite signs of coefficients in Table 1. Variation in the pooled daily nest predation rate was not related to edge complexity (FDI), neither at the local scale (1000 m circle) nor at the landscape scale (10,000 m circle).

### 3.2 Predator‐specific nest predation

At the local scale (1000 m), daily nest predation rates of most predators were higher in locations with much forest (buzzard, marten, badger, Table 1). However, daily nest predation rate by sparrowhawk at the local scale was higher in locations with little forest (Table 1). Daily nest predation rates by jay and fox did not relate to any habitat features at the local scale.

At the landscape scale (10,000 m), predator‐specific daily nest predation rates were related to different habitat features. Daily nest predation rate by buzzard decreased with a greater area of forest at the landscape scale (Table 1, Figure 1). Daily nest predation rate by marten increased with edge complexity (FDI) at the landscape scale (Table 1, Figure 1). Daily nest predation rate by badger increased with the proportion of grassland, but decreased with the proportion of arable land in the matrix (Table 1, Figure 1). Daily nest predation rates by jay, sparrowhawk, and fox were not related to habitat at the landscape scale. However, nest predation rate by jay was equally well explained by grassland in the matrix (AICc = 789.82, Table S2) as by the null model (AICc = 790.20), but the null model had fewer variables. The relationship between jay predation and grassland was positive (Coef = 0.21, SE = 0.13, Figure S7). None of the nest predation rates was related to the proportion of urban habitat in the matrix. Model predictions of total nest predation rates by predator species (i.e., proportion of nests predated on day 33 after first egg laying) are reported in Table 2.

In addition, we found that some predator species depredated wood warbler nests throughout the nesting period (i.e., egg and chick stage), while others depredated nests only during the chick stage (Figure 1, Figure S6). Buzzards, sparrowhawks, and foxes did not predate eggs, but only chicks (Figure S6). Martens and badgers depredated eggs and chicks, but their predation rate increased in the second half of the chick stage (Figure S6).

## 4 DISCUSSION

Predator‐specific wood warbler nest predation rate analyses revealed that nest predation rates varied among predators depending on the habitat. Predation rates by some predators were related to forest area, some to edge complexity, and others to the nonforested adjacent habitat matrix. The negative relationship of buzzard predation to forest area and the positive relationship of marten predation to edge complexity, both at the landscape scale, would have been missed in a pooled analysis. Likewise, the negative relationship of sparrowhawk predation to local forest area would have gone undetected. In turn, the relationships of the pooled predation rate with grassland and arable land may have been erroneously generalized if we had assessed only pooled predation rates. Previous predator‐specific nest predation studies still grouped some predators into generic categories like raptors or corvids (Benson et al., 2010; Cox et al., 2012a; DeGregorio et al., 2014; Rodewald & Kearns, 2011). We extend these studies by assessing single‐species nest predation rates of several raptor species and by investigating the relationships of nest predation rates with the habitat matrix. Next, we discuss possible mechanisms that could explain the observed relationships between the nest predation rates of wood warblers by different predators and identified associated habitat features.

Nest predation by badgers and, to some degree by jays, was higher for wood warbler nests in forest patches surrounded by a higher proportion of grassland. Although badgers are more abundant in woodland than agriculture and urban areas (Balestrieri et al., 2009; Pita et al., 2020), they often exploit different habitats (Feore & Montgomery, 1999). Badgers are more abundant in forests closer to pastoral than arable land because the availability of their main prey, the earthworm *Lumbricus terrestris*, is higher in pastures than in arable fields (da Silva et al., 1993; Kruuk et al., 1979). The higher nest predation rate by badgers in forests surrounded by grassland may thus directly reflect the higher abundance of badgers in such environments. Jays preferentially depredated passerine nests in forested areas in a landscape mosaic of forest and agriculture in southern Sweden (Andren, 1992), but our study did not support this. The high nest predation by jays in mid‐Wales (Table S1), where small forest patches are surrounded by a matrix of mostly grassland (Figure S2, S4), may have had a strong influence on the overall positive relationship between jay predation and grassland. Indeed, when omitting mid‐Wales from the analysis, the relationship between nest predation by jay and grassland became weaker (Coef = 0.05, SE = 0.16). Nevertheless, our results suggest that jays may have adapted to local forest habitat loss in mid‐Wales (Wesołowski & Fuller, 2012).

Nest predation by sparrowhawks was higher where local forest area was low. Despite typically nesting and hunting inside forests, sparrowhawks also depredate passerine nests along edges and in open habitats when leaves and ground vegetation decrease visibility in forests (Götmark & Post, 1996). In the United Kingdom, sparrowhawks also hunt in pastoral land with a rich supply of songbirds (Marquiss & Newton, 1982) and depredate house sparrows *Passer domesticus* in urban areas (Bell et al., 2010). Like predation by badgers and jays, the predation of wood warbler nests by sparrowhawks may indicate an adaptation of foraging behavior to local habitat loss. Similar adaptations to habitat loss have been observed in other species formerly inhabiting woodlands, a prominent example being the colonization of urban habitat by the European blackbird *Turdus merula* (Evans et al., 2009).

Nest predation by buzzards was negatively related to forest area at the landscape scale. Buzzards are habitat generalists and their density increases in heterogeneous landscapes due to their use of both forests and open areas to hunt various prey species (Walls & Kenward, 2020). While buzzards depend on trees for nesting, short distances between nests and nearest forest edge and prey‐rich open areas increase their reproductive success (Krüger, 2002; Sergio et al., 2005). Fragmented landscapes, where forest patches are interspersed by open fields, probably best suit their wait‐and‐strike style of hunting (Bijlsma, 1997). Moreover, buzzards are more abundant and nest‐to‐nest distance decreases in areas with small forest patches compared to large and homogeneous forests (Austin et al., 1996; Zuberogoitia et al., 2006). Thus, the negative relationship we found between nest predation by buzzards and forest area may be explained by the fragmented nature of our study regions being conducive to buzzard hunting.

In Switzerland and Germany, nest predation by pine martens was positively related to habitat edge complexity at the landscape scale. Despite the species' original specialization to forest habitat and its dependence on forests for denning (Brainerd, 1990; Bright, 1993), recent studies have shown that pine martens move slower along forest edges and hedgerows than inside forests (Pereboom et al., 2008) and have larger home ranges in fragmented landscapes (Mergey et al., 2011). According to these movement patterns, the authors suggested that pine martens preferentially forage in edge habitats (Pereboom et al., 2008) and can persist in fragmented landscapes (Mergey et al., 2011). Pine martens select young or recently felled forests rather than mature forests (Kurki et al., 1998; McNicol et al., 2020) and abundances have been increasing in agricultural areas (Balestrieri et al., 2010). Hence, pine martens seem well adapted to habitat fragmentation at the landscape level and may therefore exert high predation pressure on nests of wood warblers and possibly other ground‐nesting species in fragmented forest landscapes.

Wood warbler nest predation by foxes was not related to any of the habitat features we examined. Foxes are habitat generalists and able to access food resources in different habitats; in small or large forests, fragmented or continuous landscapes, and agriculture or urban areas (Jędrzejewski & Jędrzejewska, 1992; Webbon et al., 2004). Although a previous study showed a positive relationship of fox abundance with agriculture and a negative relationship with old growth forest (Kurki et al., 1998), it is conceivable that predation pressure from generalist predators can be independent of habitat type.

Variation in species‐specific nest predation rates is likely also related to the relative abundance of different predator species within the same study region or to variation in abundance of the same species between different regions/habitats. For example, there is some indication that buzzard populations have been increasing in the United Kingdom (Harris et al., 2021) and in Switzerland (Knaus et al., 2020), but have been decreasing in Germany (Gerlach et al., 2019, pers. comm. S. Trautmann) over the past 20–30 years. Also, the positive relationship between game bird release density and relative jay abundance in the United Kingdom (Pringle et al., 2019) may have led to higher jay abundances than would usually be predicted at the breeding range scale. Assessment of predator densities is inherently difficult but may be included in future nest predation studies.

From a management perspective, it would also be important to investigate the extent to which nest predation may be compensatory. Reduction or absence of one nest predator species may lead to increased nest predation rates by other species with different foraging behavior (Ellis‐Felege et al., 2012; Smith et al., 2010). For example, predators like ants do usually depredate chicks because intact eggs are inaccessible to ants (Staller et al., 2005). If nests are still available during the chick stage, for example, due to egg‐predator removal, these nests may be predated by predators specialized in chick predation. Here and in other studies (Benson et al., 2010; Rodewald & Kearns, 2011), raptors were shown to preferentially depredate nests at the chick stage, possibly due to raptors' visual foraging technique being more efficient during the chick stage when adult provisioning activity is increased (Benson et al., 2010; Weidinger, 2010). Hence, the absence of martens or a potential removal of badgers (e.g., in the United Kingdom), both predators depredating eggs and chicks, may not lead to increased nesting success in wood warblers if raptor predation at the chick stage has a compensatory effect.

## 5 CONCLUSIONS

Our results on wood warbler nest predation rates corroborate existing knowledge; for instance that nest predation by raptors increases in fragmented landscapes with little forest (buzzard) or close to agricultural edges (e.g., red‐shouldered hawk, Benson et al., 2010). Some of our results differ from other studies; for example, nest predation by jays was positively related to the proportion of grassland in our study, but was negatively associated with grassland elsewhere (Andren, 1992). The differences among studies and the variation in habitat associations of wood warbler nest predation by different predator species highlight that generalizations about the association of nest predation and habitat should be made with care. This is because habitat associations depend on the predator species involved and may vary between populations of the same species.

To adjust to spatial variation in habitat availability and quality, animals exhibit varying degrees of habitat association (Mayr, 1963), and predators that mainly forage in woodlands in some parts of their distribution can adapt foraging behavior in response to habitat loss elsewhere (Evans et al., 2009; Wesołowski & Fuller, 2012). However, it is questionable if adaptations of foraging patterns by predators to habitat loss leads to decreased nest success in wood warblers and other ground‐nesting birds, as nest failure rates of wood warblers in Western Europe are similar to those in Eastern Europe, where deforestation is less severe (Maag et al., 2022). As ground‐nesting birds, wood warblers are well adapted to high nest predation rates, but when there are other potential pressures on a population, high nest predation rates may become detrimental to avian populations (Newton, 1998). Hence, it is important to understand the habitat associations of predator‐specific nest predation rates, rather than those of pooled predation rates, when assessing the impact of habitat on nesting success. Otherwise, important habitat features that influence nest predation rates may be overlooked, potentially leading to misinformed conservation efforts.

## AUTHOR CONTRIBUTIONS

**Nino Maag:** Conceptualization (lead); data curation (lead); formal analysis (lead); visualization (lead); writing – original draft (lead). **John W. Mallord:** Investigation (equal); writing – review and editing (equal). **Malcolm D. Burgess:** Investigation (equal); writing – review and editing (equal). **Shannon Lüpold:** Investigation (equal); writing – review and editing (equal). **Andrew Cristinacce:** Investigation (equal); writing – review and editing (supporting). **Raphaël Arlettaz:** Resources (supporting); writing – review and editing (supporting). **Sandro Carlotti:** Investigation (equal); writing – review and editing (supporting). **Tony M. Davis:** Investigation (equal); writing – review and editing (supporting). **Alex Grendelmeier:** Investigation (equal); writing – review and editing (supporting). **Christopher J. Orsman:** Investigation (equal). **Michael Riess:** Investigation (equal). **Pablo Stelbrink:** Investigation (equal). **Gilberto Pasinelli:** Conceptualization (supporting); funding acquisition (lead); investigation (supporting); project administration (lead); writing – review and editing (lead).

## Supporting information


Appendix S1

## Data Availability

Data supporting this manuscript are available in the vogelwarte.ch Open Repository and Archive: https://doi.org/10.5281/zenodo.7088465. Please contact the authors before using the dataset.
