# Supplementary material for: Accounting for predator species identity reveals variable relationships between nest predation rate and habitat in a temperate forest songbird
Source: Ecol Evol. 2022 Oct 12;12(10):e7411. doi: 10.1002/ece3.9411 (PMC9557003; doi:10.1002/ece3.9411)
Supplement: Supplementary file 1 — Appendix S1 [file ECE3-12-e7411-s001.pdf]

Supplementary material

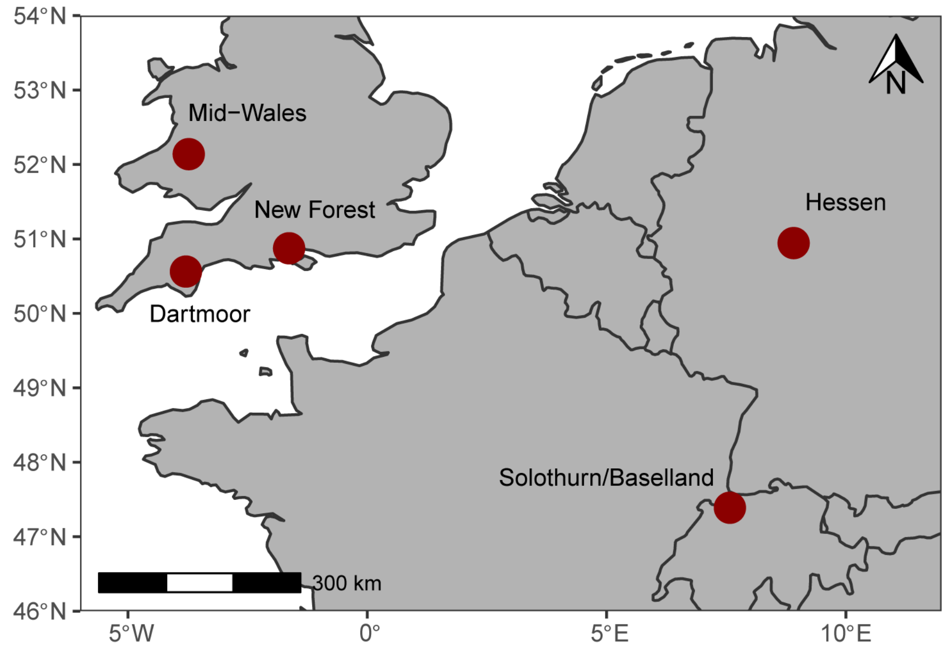

Figure S1. Locations of the five study regions (red points).

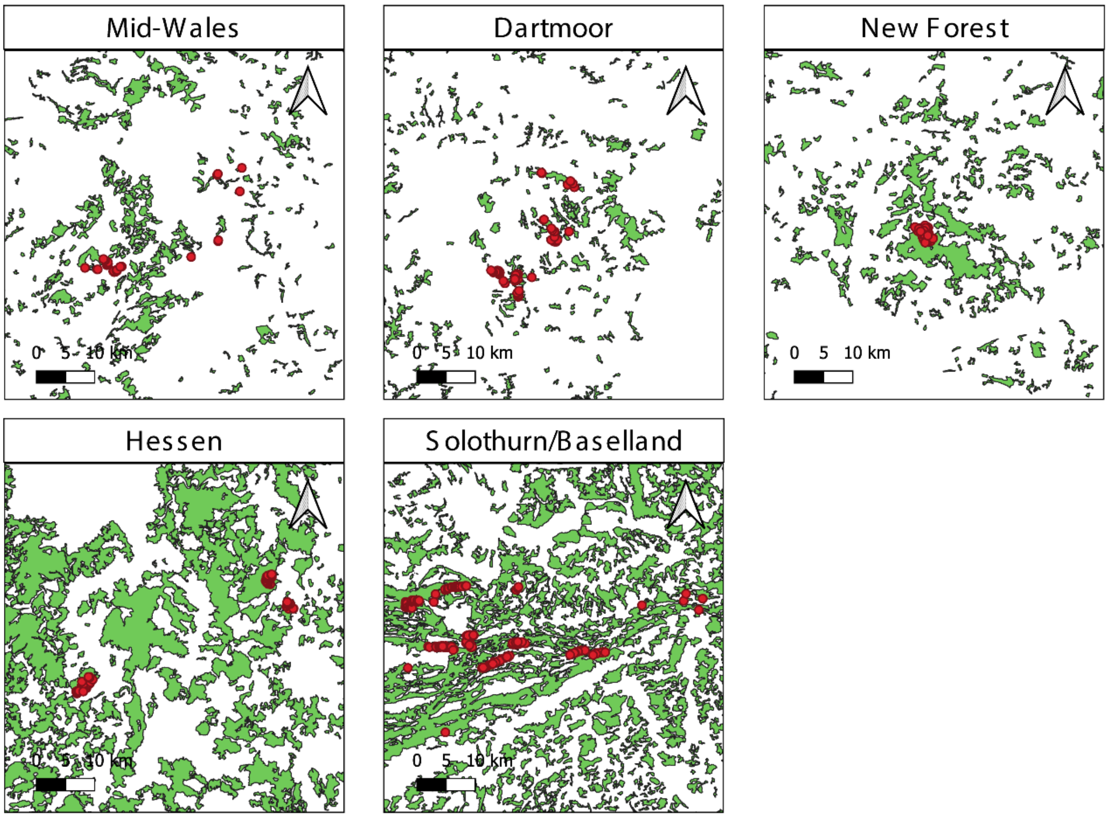

Figure S2. Forest area and fragmentation in the five study areas. Forest areas are shown in green, non-forest areas (e.g., grassland, arable land, and urban habitat) in white. Red points show wood warbler nest locations.

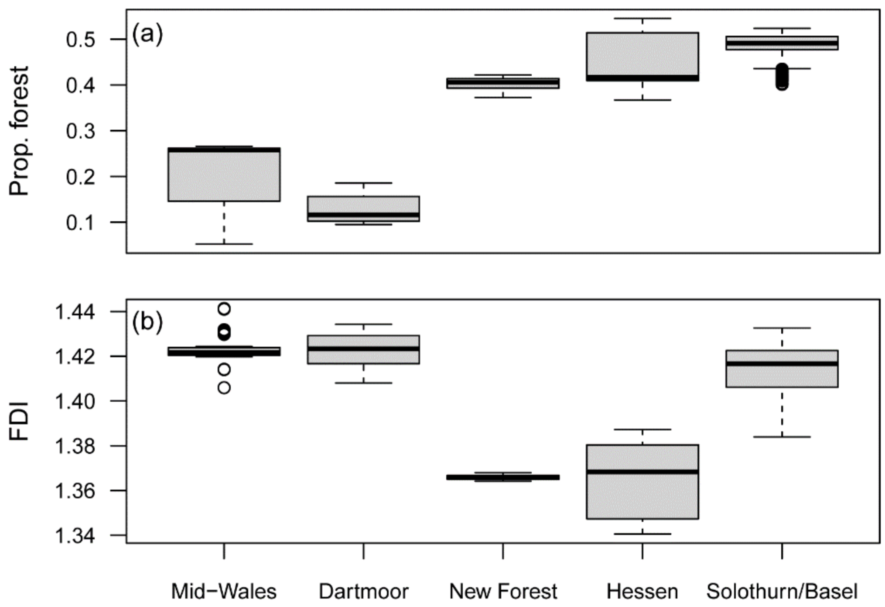

Figure S3. Forest area (a) and fractal dimension index FDI (b) at the landscape level - i.e., inside circles with a radius of 10,000 m around wood warbler nests - in the five study regions. Forest area = proportion of the cumulative forest area within the total circle area; FDI = two times the natural logarithm of the total forest edge divided by the natural logarithm of total forest area. Sample sizes: $n_{Mid\text{-}Wales}$=73 nests, $n_{Dartmoor}$=65, $n_{New\ Forest}$=45, n_{Hessen}=89, $n_{Solothurn/Basel}$=287.

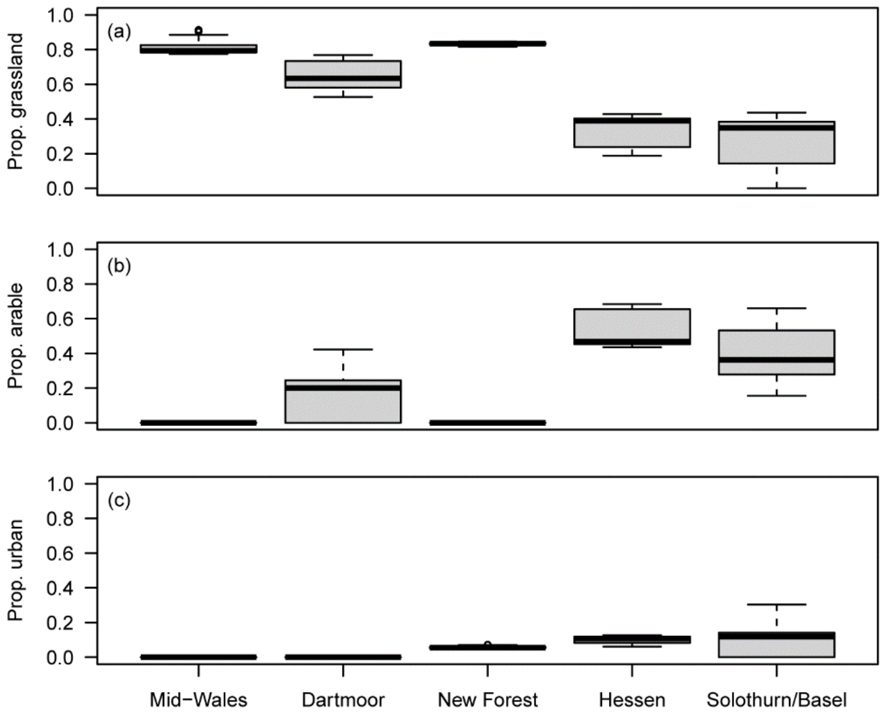

Figure S4. Proportion of grassland (a), arable (b), and urban area (c) in the matrix adjacent to forest areas with wood warbler nests in the five study regions. Grassland mostly consisted of pasture and natural grassland, and included small fractions of heathland, sparse vegetation, and peat bogs; arable area consisted of arable fields and mosaics of small cultivated land parcels with different cultivation types; urban area consisted of discontinuous urban habitat. Sample sizes: $n_{Mid\text{-}Wales}$=73 nests, $n_{Dartmoor}$=65, $n_{New\ Forest}$=45, n_{Hessen}=89, $n_{Solothurn/Basel}$=287.

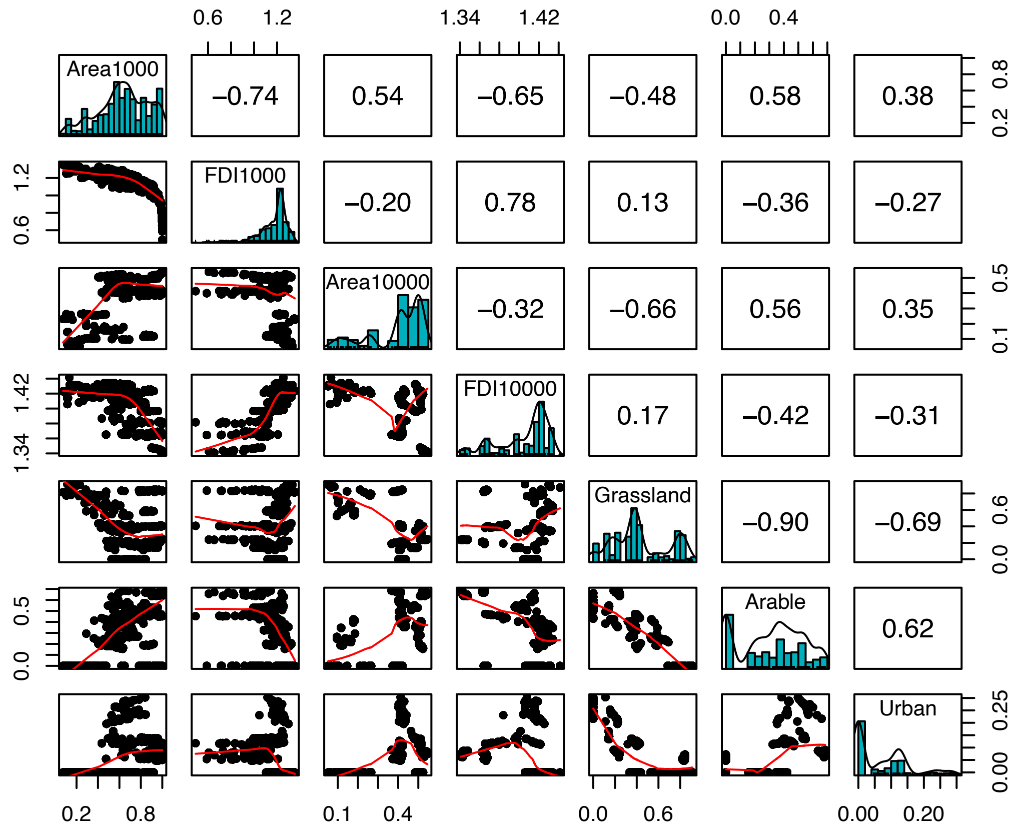

Figure S5. Pair-wise comparison between all fixed effects used in the predation rate analyses. Pearson correlation coefficients and scatterplots are shown for each pair of fixed effects. Sample size: n=516 nests (559 total nests – 43 nests lost to non-predation related causes).

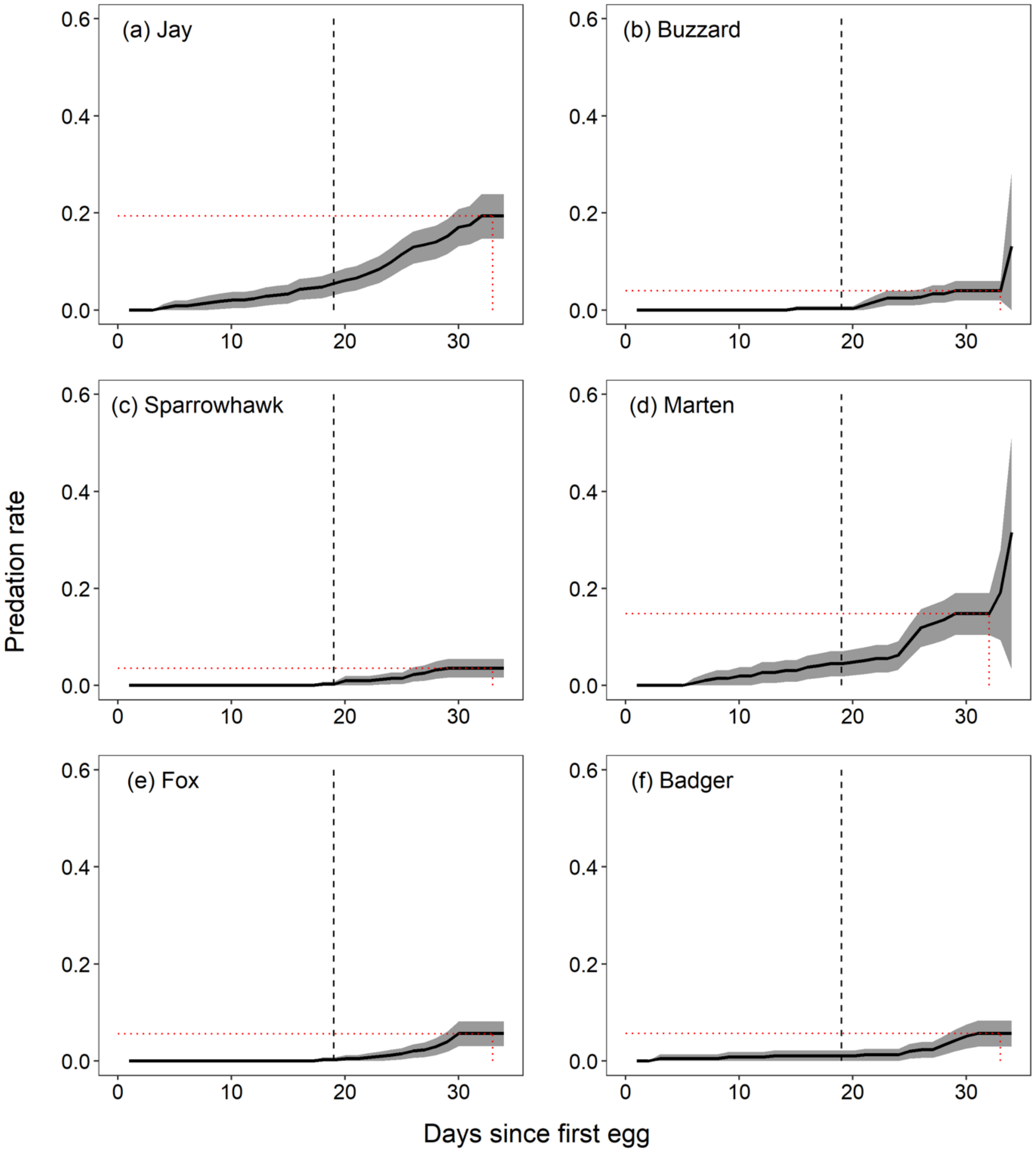

Figure S6. Predator-specific daily predation rates of wood warbler nests. Shown are daily nest predation rates and 95% confidence intervals starting at the first egg laying date. Vertical black dashed lines indicate the average hatching date, dividing the nesting period into egg and chick stage. Red dotted lines indicate the overall nest predation rate by predators 33 days after first egg laying, the average nesting period in wood warblers (except for martens, where we chose 32 days due to the large confidence intervals after day 32). Sample sizes (including predated and successful nests): n_{Jay}=368, $n_{Buzzard}$=317, $n_{Sparrowhawk}$=314, n_{Marten}=297 (excl. UK study areas), n_{Fox}=319, n_{Badger}=319.

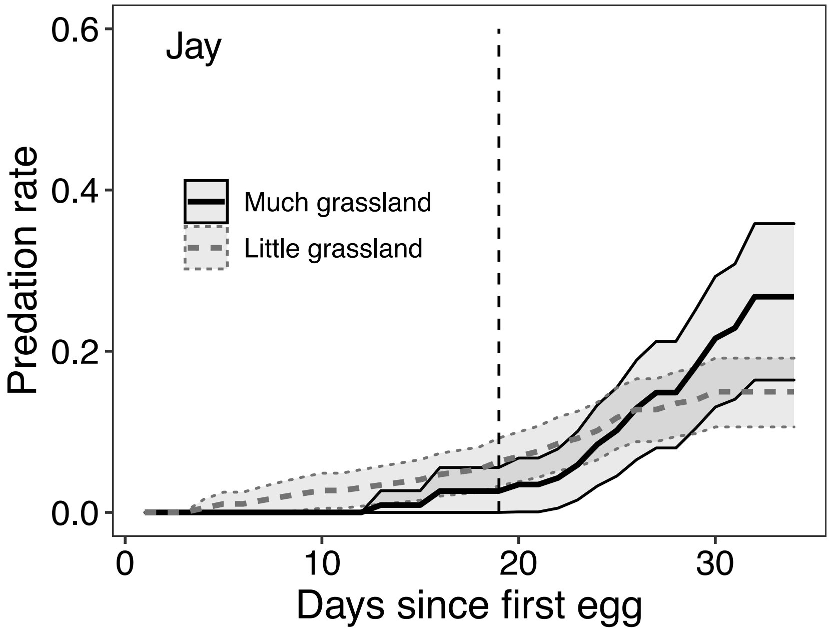

Figure S7. Daily predation rates of wood warbler nests by jays in relation to grassland in the adjacent matrix (little ≤ mean, much > mean). Vertical black dashed line indicates the average hatching date. Shown are daily nest predation rate and 95% confidence interval starting at the first egg laying date. Sample size (including predated and successful nests): n_{Jay}=368.

Table S1. Number of wood warbler nests predated by different species as identified by nest cameras in the five study regions mid-Wales (MW), Dartmoor (DM), New Forest (NF), Hessen (HE), and Solothurn-Baselland (SB). Brackets show the percentage of nests, compared to all nests in a study region, that were predated by a specific predator, failed due to other reasons, or survived.

	Predator	MW	DM	NF	HE	SB	Total
Bird	Eurasian jay *Garrulus glandarius*	18 (24.7%)	5 (7.7%)	5 (11.1%)	13 (14.6%)	27 (9.3%)	68 (12.2%)
	Common buzzard *Buteo buteo*	4 (5.5%)	8 (12.3%)	1 (2.2%)	1 (1.1.%)	2 (0.7%)	16 (2.9%)
	Eurasian sparrowhawk *Accipiter nisus*	2 (2.7%)	4 (6.2%)	1 (2.2%)	0	6 (2.1%)	13 (2.3%)
	Tawny owl *Strix aluco*	0	0	2 (4.4%)	0	3 (1.0%)	5 (0.9%)
	Great spotted woodpecker *Dendrocopos major*	1 (1.4%)	0	0	1 (1.1.%)	1 (0.3%)	3 (0.5%)
	Honey buzzard *Pernis apivorus*	0	0	0	0	1 (0.3%)	1 (0.2%)
	Blackbird *Turdus merula*	0	0	0	0	1 (0.3%)	1 (0.2%)
Mammal	Pine marten *Martes martes*	0	0	0	2 (2.2%)	39 (13.5%)	41 (7.3%)
	Red fox *Vulpes vulpes*	1 (1.4%)	1 (1.5%)	2 (4.4%)	2 (2.2%)	12 (4.2%)	18 (3.2%)
	European badger *Meles meles*	1 (1.4%)	3 (4.6%)	4 (8.9%)	3 (3.4%)	7 (2.4%)	18 (3.2%)
	Various rodents *Apodemus, Myodes, Glis, Microtus, Mus, Rattus*	0	3 (4.6%)	2 (4.4%)	2 (2.2%)	7 (2.4%)	14 (2.5%)
	Racoon *Procyon lotor*	0	0	0	6 (6.7%)	0	6 (1.1%)
	Grey squirrel *Sciurus carolinensis*	0	1 (1.5%)	2 (4.4%)	0	0	3 (0.5%)
	European hedgehog *Erinaceus europaeus*	0	0	0	3 (3.4%)	0	3 (0.5%)
	Wildboar *Sus scrofa*	0	0	0	1 (1.1.%)	1 (0.3%)	2 (0.4%)
	Least weasel *Mustela nivalis*	0	2 (3.1%)	0	0	0	2 (0.4%)
	Red squirrel *Sciurus vulgaris*	0	0	0	0	1 (0.3%)	1 (0.2%)
	Cat *Felis catus*	0	0	0	0	1 (0.3%)	1 (0.2%)
	Dog *Canis familiaris*	0	0	1 (2.2%)	0	0	1 (0.2%)
Other pred.	Unknown	5 (6.8%)	5 (7.7%)	8 (17.8%)	5 (5.6%)	11 (3.8%)	34 (6.1%)
	Slug *Arion sp.*	0	0	0	0	2 (0.7%)	2 (0.4%)
	European adder *Vipera berus*	0	0	2 (4.4%)	0	0	2 (0.4%)
Other fail.	E.g., desertion, trampling	2 (2.7%)	13 (20.0%)	3 (6.7%)	7 (7.9%)	18 (6.2%)	43 (7.7%)
Fledged	Min. one chick fledged	39 (53.4%)	20 (30.8%)	12 (26.7%)	43 (48.3%)	149 (51.6%)	263 (47.0%)

Table S2. Results from model selection based on Akaike's information criterion (subset of models with ΔAICc<2) for each of the seven Cox hazard models including grassland. AICc = AIC for small sample sizes, delta = difference to model with lowest AICc. In bold are models with fewer number of parameters, which we identified as the most parsimonious explaining hazard rates. Model variables: forest area and FID at the 1,000 m and 10,000 m scales, respectively, and urban habitat and grassland in the adjacent matrix.

Model	AICc	delta
Pooled (n=516):		
Area1,000+FDI10,000+Grassland	2880.73	0.00
**Area1,000+Grassland**	**2880.85**	**0.12**
Area1,000+Grassland+Urban	2882.12	1.38
Area1,000+Area10,000+FDI10,000+Grassland	2882.35	1.61
Area1,000+Urban	2882.44	1.71
Area1,000+FDI10,000+Grassland+Urban	2882.58	1.85
Area1,000+Area10,000+Grassland	2882.68	1.95
Jay (n=368):		
Grassland+Urban	789.40	0.00
Grassland	789.82	0.42
**Null**	**790.20**	**0.80**
Area10,000+Grassland+Urban	791.05	1.65
FDI10,000+Grassland+Urban	791.07	1.67
Area1,000+Grassland+Urban	791.14	1.74
FDI10,000+Grassland	791.37	1.97
Buzzard (n=317):		
**Area1,000+Area10,000**	**162.28**	**0.00**
Area1,000+Area10,000+FDI10,000	163.72	1.44
Area1,000+Area10,000+Urban	164.00	1.72
Area1,000+Area10,000+Grassland	164.18	1.89
Sparrowhawk (n=314):		
Area1,000	149.57	0.00
**Null**	**151.05**	**1.48**
Area1,000+Grassland	151.24	1.66
Area1,000+Area10,000	151.31	1.74
Area1,000+Urban	151.44	1.87
Area1,000+FDI10,000	151.48	1.91
Marten (n=297):		
Area1,000+Area10,000+FDI10,000+Grassland+Urban	398.53	0.00
**Area1,000+FDI10,000**	**399.21**	**0.68**
Area1,000+FDI10,000+Grassland	399.96	1.42
Area10,000+FDI10,000+Grassland+Urban	400.20	1.67
Area1,000+FDI10,000+Urban	400.53	2.00
Fox (n=319):		
**Null**	**209.29**	**0.00**
Grassland	209.33	0.04
Area10,000	209.45	0.17
Area1,000	210.28	0.99
Urban	210.44	1.16
FDI10,000	210.74	1.45
FDI10,000+Grassland	210.94	1.66
Grassland+Urban	210.97	1.69
Area10,000+Grassland	211.04	1.75
Area1,000+Grassland	211.18	1.89
Area10,000+FDI10,000	211.21	1.92
Badger (n=319):		
**Area1,000+Grassland**	**192.46**	**0.00**
Area1,000+Grassland+Urban	193.85	1.39
Area1,000+Area10,000+Grassland	193.96	1.50
Area1,000+Area10,000	194.04	1.58
Area1,000+FDI10,000+Grassland	194.26	1.80

Table S3. Results from model selection based on Akaike's information criterion (subset of models with ΔAICc<2) for each of the seven Cox hazard models including arable land. AICc = AIC for small sample sizes, delta = difference to model with lowest AICc. In bold are models with fewer number of parameters, which we identified as the most parsimonious explaining hazard rates. Model variables: forest area and FID at the 1,000 m and 10,000 m scales, respectively, and urban habitat and arable land in the adjacent matrix.

Model	AICc	delta
Pooled (n=516):		
**Arable+Area1,000**	**2880.95**	**0.00**
Arable+Area1,000+FDI10,000	2881.97	1.02
Arable+Area1,000+Urban	2881.99	1.05
Area1,000+Urban	2882.44	1.50
Arable+Area1,000+Area10,000	2882.77	1.83
Jay (n=368):		
**Null**	**790.20**	**0.00**
Arable	791.21	1.01
Area10,000	791.46	1.26
Area1,000	791.51	1.31
FDI10,000	791.64	1.44
Urban	791.68	1.49
Arable+Urban	792.18	1.99
Buzzard (n=317):		
**Area1,000+Area10,000**	**162.28**	**0.00**
Arable+Area1,000+Area10,000	163.65	1.37
Area1,000+Area10,000+FDI10,000	163.72	1.44
Area1,000+Area10,000+Urban	164.00	1.72
Sparrowhawk (n=314):		
Arable+FDI10,000+Urban	148.63	0.00
**Area1,000**	**149.57**	**0.94**
Arable+Area10,000+FDI10,000+Urban	149.81	1.18
Arable	150.30	1.67
Arable+Area1,000	150.31	1.67
Arable+Area1,000+FDI10,000+Urban	150.42	1.79
Arable+FDI10,000	150.60	1.96
Marten (n=297):		
Arable+Area1,000+FDI10,000	398.76	0.00
**Area1,000+FDI10,000**	**399.21**	**0.46**
Arable+Area1,000+Area10,000+FDI10,000	399.56	0.81
Area1,000+FDI10,000+Urban	400.53	1.78
Arable+Area1,000+FDI10,000+Urban	400.75	2.00
Fox (n=319):		
**Null**	**209.29**	**0.00**
Area10,000	209.45	0.17
Arable	209.89	0.60
Area1,000	210.28	0.99
Urban	210.44	1.16
FDI10,000	210.74	1.45
Area10,000+FDI10,000	211.21	1.92
Arable+Area10,000	211.22	1.94
Badger (n=319):		
**Arable+Area1,000**	**192.64**	**0.00**
Arable+Area1,000+Area10,000	193.70	1.06
Area1,000+Area10,000	194.04	1.40
Arable+Area1,000+Urban	194.35	1.71
Arable+Area1,000+FDI10,000	194.60	1.96
